# Tracking COVID-19 in Europe: Infodemiology Approach

**DOI:** 10.2196/18941

**Published:** 2020-04-20

**Authors:** Amaryllis Mavragani

**Affiliations:** 1 Department of Computing Science and Mathematics Faculty of Natural Sciences University of Stirling Stirling United Kingdom

**Keywords:** big data, coronavirus, COVID-19, infodemiology, infoveillance, Google Trends

## Abstract

**Background:**

Infodemiology (ie, information epidemiology) uses web-based data to inform public health and policy. Infodemiology metrics have been widely and successfully used to assess and forecast epidemics and outbreaks.

**Objective:**

In light of the recent coronavirus disease (COVID-19) pandemic that started in Wuhan, China in 2019, online search traffic data from Google are used to track the spread of the new coronavirus disease in Europe.

**Methods:**

Time series from Google Trends from January to March 2020 on the Topic (Virus) of “Coronavirus” were retrieved and correlated with official data on COVID-19 cases and deaths worldwide and in the European countries that have been affected the most: Italy (at national and regional level), Spain, France, Germany, and the United Kingdom.

**Results:**

Statistically significant correlations are observed between online interest and COVID-19 cases and deaths. Furthermore, a critical point, after which the Pearson correlation coefficient starts declining (even if it is still statistically significant) was identified, indicating that this method is most efficient in regions or countries that have not yet peaked in COVID-19 cases.

**Conclusions:**

In the past, infodemiology metrics in general and data from Google Trends in particular have been shown to be useful in tracking and forecasting outbreaks, epidemics, and pandemics as, for example, in the cases of the Middle East respiratory syndrome, Ebola, measles, and Zika. With the COVID-19 pandemic still in the beginning stages, it is essential to explore and combine new methods of disease surveillance to assist with the preparedness of health care systems at the regional level.

## Introduction

In December 2019, Chinese researchers identified a novel coronavirus in humans that caused acute respiratory syndrome—officially called coronavirus disease (COVID-19) as of February 11, 2020 [1]. China reported its first death on January 11, 2020, and Wuhan in the Hubei province, which was identified as the epicenter of the epidemic, was cut off by Chinese authorities on January 23, 2020 [2].

COVID-19 quickly surpassed the death toll of the severe acute respiratory syndrome (SARS) pandemic on February 9, 2020 [2]. The virus had already spread to several other Chinese regions, quickly affecting many neighboring countries as well, like the Philippines and South Korea [2]. Several cases of COVID-19 were reported throughout Europe over the next days without causing any regional epidemic at the time; although this did not last long, with Italy having its first death on February 21, 2020 [3], which in a short time spread to all European countries, resulting in the World Health Organization declaring it a pandemic on March 11, 2020 [4].

**Figure 1:**
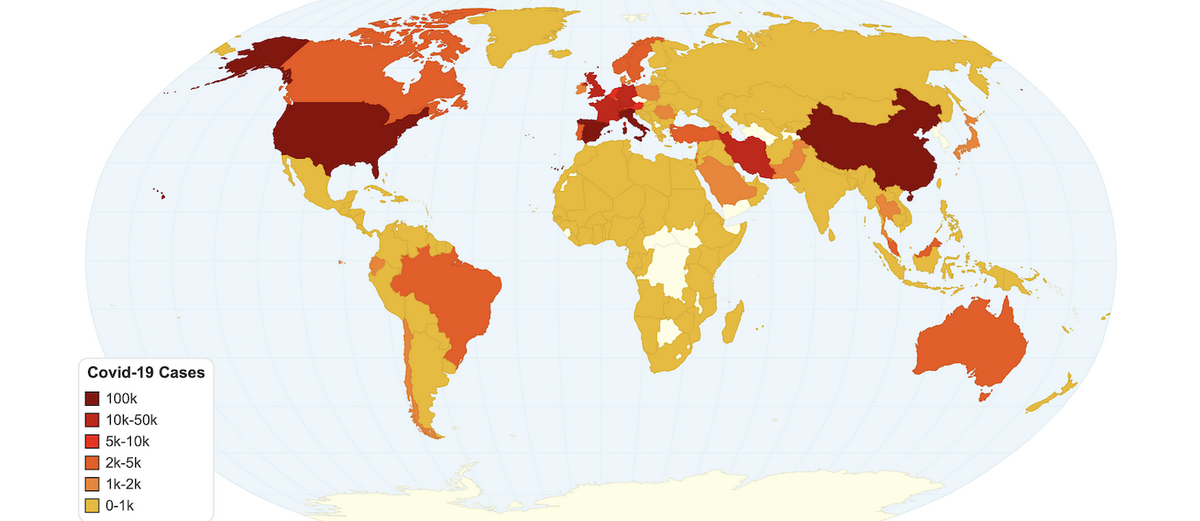
Worldwide heat map for total COVID-19 cases by country (as of March 25, 2020).

**Figure 2:**
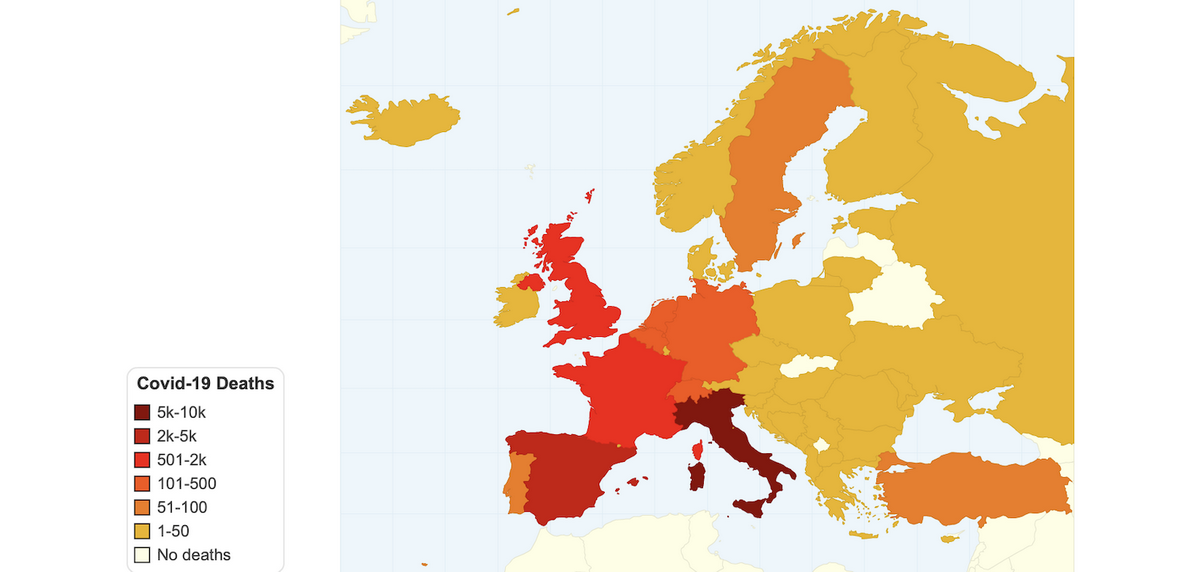
European heat map for total COVID-19 deaths by country (as of March 25th, 2020).

As of March 25, 2020, COVID-19 cases have surpassed 471,000 worldwide, with more than 335,000 still active, and with more than 21,000 deaths. The country with the most confirmed COVID-19 cases is the United States with 81,864, almost half of which are in the state of New York. Italy is the most affected country in number of deaths as of March 25, with 74,386 cases and 7503 deaths. Lombardy, the origin of the Italy epidemic, is the most affected region, followed by Emilia-Romagna, Veneto, Piedmont, Marche, Tuscany, and Liguria. In Europe, Spain is unfortunately following Italy’s curve, with 49,515 cases and 3647 deaths. Both countries have surpassed China’s 3287 reported COVID-19 death toll. France and Germany are also facing a difficult situation, with more than 29,155 and 43,646 confirmed cases, respectively. All European countries have COVID-19 cases, and most countries have at least one death. However, there is a clear geographical distribution of COVID-19 cases in Europe, with central and southwest Europe being the most affected. Figure 1 depicts the current situation in COVID-19 cases worldwide up to March 25, 2020, while Figure 2 shows the COVID-19 (total cumulative, not per capita) deaths by country up to March 25, 2020. All data on COVID-19 cases and deaths were retrieved from Worldometer [5].

Italy is the first country facing serious issues and a large number of deaths due to COVID-19 in Europe, followed by Spain, France, Germany, and the United Kingdom [5]. The main issue in all affected countries is that of the health systems’ capabilities and performance. Toward this direction and based on early Italian data about the spread of the disease, all European countries have taken measures aiming at “flattening the curve” [6], meaning to spread the cases—and, consequently, the patients that need to be admitted to the intensive care unit—over a longer period of time.

Said measures mainly consist of flight restrictions, borders closing, shutting down cafes and restaurants, closing of schools, and self-isolation at first and restriction of movement afterwards, with a total lockdown being the last resort, which has unfortunately been taken in several cases, like that of Lombardy and Spain. The United Kingdom and the Netherlands followed a different approach at first, despite the Imperial College’s Response Team’s reports led by Prof Ferguson [7-9], with many claiming that they were aiming at herd immunity, which also posed several ethical concerns. Even these two countries, however, resorted to some measures and restrictions at the end [10,11].

As Gunther Eysenbach, who first proposed the concept of infodemiology (ie, information epidemiology [12-14]), suggested during the SARS pandemic, the use of population health technologies such as the internet can assist with the detection of diseases during an early stage [15]. Given the serious impact of the novel coronavirus and toward the direction of using new methods and approaches for the nowcasting and forecasting of this pandemic, in this paper, Google Trends data are used to explore the relationship between online interest in COVID-19 and cases and deaths in severely affected European countries (ie, Italy, Spain, France, Germany, and the United Kingdom). During these times, infodemiology metrics, especially if combined with traditional data, can be an integral part of the surveillance of the virus at the regional level.

## Methods

Data from Google Trends [16] are normalized and retrieved online in .csv format. Note that data may slightly vary based on the time of retrieval. Time series from Google Trends for various time intervals from January to March 2020 on the Topic (Virus) of “Coronavirus” are used, combined with official data on COVID-19 cases and deaths retrieved from Worldometer [5]. The aim is to track the spread of the disease in the European countries that have been affected the most (ie, Italy, Spain, France, Germany, and the United Kingdom). Regional analysis is performed in Italy (data from the Ministry of Health [17]), and the Pearson correlation coefficients between COVID-19 cases and deaths and Google Trends time series are calculated. The Topic of “Coronavirus” was selected instead of the “COVID-19” search term, as the latter was not widely used up to the point of the analysis.

For the general worldwide interest and correlation analysis, the period was set from January 22 to March 17, 2020, while for the rest of the European countries it was set from February 15 to March 17. For the detailed European countries’ correlation analysis, case and death data from March 2 to 17 were used. A new data set was retrieved for each time frame, which matched the official COVID-19 case data. The default “All categories” and “Web search” were selected. Note that each country, region, and county were examined individually, and no comparisons between countries in COVID-19 data or Google data were made. The heat maps are based on absolute numbers for COVID-19 cases and deaths, and not according to the respective population. The methodology was designed based on the Google Trends methodology framework in infodemiology and infoveillance [18].

## Results

Table 1 consists of the Pearson correlation coefficients (*r*) between Google Trends data and the respective categories of total (cumulative) and daily cases and deaths (where applicable), worldwide (January 22 to March 17) and in the five most affected European countries (February 15 to March 17) (ie, Italy, Spain, France, Germany, and the United Kingdom). Note that for the total worldwide cases excluding China, the Pearson correlation coefficient (*r*) is .9430, with *P*<.001.

**Table 1:** Pearson correlation coefficients (*r*) between Google Trends and COVID-19 data.

Variables	Worldwide			Italy			Spain			France			Germany			United Kingdom	
	*r*	*P* value	*r*		*P* value	*r*		*P* value	*r*		*P* value	*r*		*P* value	*r*		*P* value
Total cases	0.8293	<.001	0.3301		.07	0.7363		<.001	0.8709		<.001	0.674		<.001	0.8956		<.001
Total deaths	0.8917	<.001	0.2837		.12	Ν/Α^a^		Ν/Α	0.8542		<.001	Ν/Α		Ν/Α	Ν/Α		Ν/Α
Daily new cases	0.7575	<.001	0.3931		.03	0.8342		<.001	Ν/Α		Ν/Α	Ν/Α		Ν/Α	0.8479		<.001
Daily new deaths	0.8536	<.001	0.3474		.05	Ν/Α		Ν/Α	0.8554		<.001	Ν/Α		Ν/Α	Ν/Α		Ν/Α

^a^N/A: not applicable.

**Figure 3:**
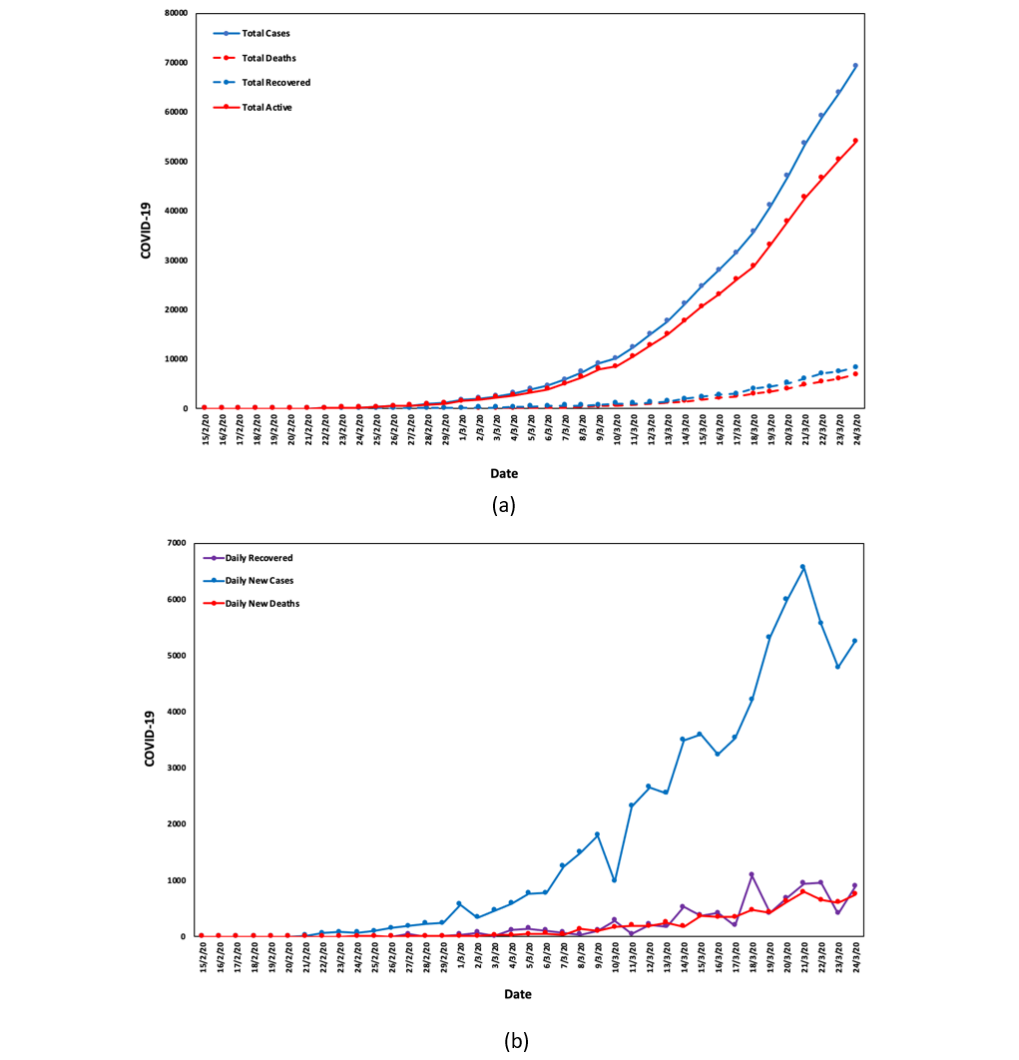
(a) Cumulative and (b) daily cases, recoveries, and deaths (Italy; February 15-March 24).

Based on the results, high statistical significance was observed for the correlations between Google and COVID-19 data for all countries and all applicable categories, apart from Italy, where Google data and COVID-19 total deaths were not correlated. In Italy, total cases and daily deaths were statistically significant but with lower significance, which is not in line with the results for the rest of the countries. The latter could be due to Italy’s current special circumstances; it is the first European country to experience such severe consequences from COVID-19 and is further along the line compared with the rest of the countries. Figure 3 depicts the cumulative and daily cases, recoveries, and deaths from February 15 to March 24 in Italy.

Thus, what is essential at this point is to examine if there had been periods for which COVID-19 cases and deaths in Italy correlated with Google query data. The following time frames were selected: March 2-9, March 2-10, March 2-11, March 2-12, March 2-13, March 2-13, March 2-14, March 2-15, March 2-16, and March 2-17.

Table 2 consists of the correlations between Google Trends data and cases, deaths, daily new cases, and daily new deaths in Italy for the aforementioned time frames. Tables 3-4 consist of the individual regions’ correlations between COVID-19 cases and Google data.

**Table 2:** Pearson correlation coefficients (*r*) between COVID-19 cases and deaths and Google Trends data in Italy.

Time frames	Cases		Deaths			Daily Cases^a^			Daily Deaths^a^		
	*r*	*P* value		*r*	*P* value		*r*	*P* value		*r*	*P* value
March 2-9	0.9484	<.001		0.9336	<.001		0.9574	<.001		0.8097	.02
March 2-10	0.9157	<.001		0.8593	.003		0.8796	.002		0.7901	.01
March 2-11	0.8951	<.001		0.8261	.003		0.8473	.002		0.7979	.006
March 2-12	0.7942	.004		0.7279	.01		0.7644	.006		0.7792	.005
March 2-13	0.6357	.03		0.5605	.06		0.6768	.02		0.6401	.03
March 2-14	0.5067	.08		0.4537	.12		0.5394	.06		0.6223	.02
March 2-15	0.4417	.11		0.3949	.16		0.4828	.08		0.5071	.06
March 2-16	0.2944	.29		0.2410	.39		0.4065	.13		0.3678	.18
March 2-17	0.1588	.56		0.1036	.70		0.0388	.89		0.2624	.33

^a^Refers to daily new cases and deaths.

**Table 3:** Pearson correlation coefficients (*r*) between COVID-19 cases and Google Trends data in the 20 Italian regions for March 2-9; March 2-10; March 2-11; March 2-12.

Region	March 2-9		March 2-10		March 2-11		March 2-12	
	*r*	*P* value	*r*	*P* value	*r*	*P* value	*r*	*P* value
Lombardia	0.8987	.002	0.8876	.001	0.8625	.001	0.7502	.008
Emilia-Romagna	0.9017	.002	0.8839	.002	0.8798	<.001	0.8292	.002
Veneto	0.9117	.002	0.9230	<.001	0.9139	<.001	0.7960	.003
Piedmont	0.9494	<.001	0.8690	.002	0.8545	.002	0.7537	.007
Marche	0.8770	.005	0.8301	.006	0.8384	.002	0.7551	.007
Liguria	0.8739	.002	0.8451	.004	0.8042	.005	0.6810	.02
Campania	0.9506	<.001	0.9289	<.001	0.9175	<.001	0.8616	<.001
Toscana	0.9073	.002	0.8279	.006	0.8274	.003	0.7529	.007
Lazio	0.9458	<.001	0.9243	<.001	0.8883	<.001	0.7712	.005
Friuli	0.9310	<.001	0.9407	<.001	0.9284	<.001	0.8493	<.001
Trento	0.8722	.005	0.7934	.01	0.7364	.02	0.6978	.02
Apulia	0.9092	.002	0.9005	<.001	0.8573	.002	0.7894	.004
Sicily	0.9725	<.001	0.9691	<.001	0.9510	<.001	0.8604	<.001
Abruzzo	0.8720	.005	0.8523	.004	0.8685	.001	0.6261	.04
Umbria	0.8775	.004	0.8636	.003	0.8158	.004	0.7104	.01
Aosta	0.8704	.005	0.8179	.007	0.7870	.007	0.5679	.07
Sardinia	0.9170	.001	0.9047	<.001	0.7676	.009	0.7268	.01
Calabria	0.9054	.002	0.9004	<.001	0.8413	.002	0.7197	.01
Molise	0.7101	.048	0.7382	.02	0.7160	.02	0.6764	.02
Basilicata	0.8881	.003	0.7884	.01	0.8306	.003	0.8278	.002

**Table 4:** Pearson correlation coefficients (*r*) between COVID-19 cases and Google Trends data in the 20 Italian regions for March 2-13; March 2-14; March 2-15; March 2-16; March 2-17.

Region	March 2-13			March 2-14			March 2-15			March 2-16			March 2-17	
	*r*	*P* value	*r*		*P* value	*r*		*P* value	*r*		*P* value	*r*		*P* value
Lombardia	0.5864	.045	0.4216		.15	0.348		.22	0.1676		.55	0.0693		.80
Emilia-Romagna	0.6471	.02	0.5013		.08	0.442		.11	0.2773		.32	0.1406		.60
Veneto	0.6557	.02	0.4931		.09	0.4900		.08	0.3542		.20	0.2286		.39
Piedmont	0.5599	.06	0.3969		.18	0.3181		.27	0.1341		.63	0.0329		.90
Marche	0.4817	.11	0.2615		.39	0.1687		.56	–0.0869		.76	–0.1932		.47
Liguria	0.5682	.05	0.4111		.16	0.3145		.27	0.2237		.42	0.1166		.67
Campania	0.7073	.01	0.5285		.06	0.4668		.09	0.2611		.35	0.0789		.77
Toscana	0.5822	.047	0.4447		.13	0.396		.16	0.2228		.43	0.115		.67
Lazio	0.4665	.13	0.3157		.29	0.27		.35	0.0683		.81	–0.0746		.78
Friuli	0.6211	.03	0.4791		.097	0.4274		.13	0.2872		.30	0.1774		.51
Trento	0.4813	.11	0.3592		.23	0.2652		.36	0.0553		.85	–0.0388		.89
Apulia	0.6426	.02	0.4421		.13	0.3555		.21	0.2495		.37	0.0419		.88
Sicily	0.7720	.003	0.7055		.007	0.6291		.02	0.5398		.04	0.4332		.09
Abruzzo	0.5535	.06	0.4495		.12	0.4362		.12	0.2808		.31	0.1717		.53
Umbria	0.6088	.04	0.4299		.14	0.3501		.21	0.2063		.46	0.0649		.81
Aosta	0.5123	.09	0.3779		.20	0.2761		.34	0.1942		.49	0.114		.67
Sardinia	0.6188	.03	0.5551		.049	0.5808		.03	0.4049		.13	0.3125		.24
Calabria	0.6272	.03	0.5594		.047	0.5310		.05	0.4234		.12	0.2467		.36
Molise	0.7222	.008	0.4785		.098	0.4498		.12	0.3883		.15	0.232		.39
Basilicata	0.7522	.005	0.7239		.005	0.6253		.02	0.5945		.02	0.4291		.097

As is evident, the strength of the correlation decreases as the time frame includes days when the disease was already widespread, both for cumulative and daily cases and deaths. This is due to the critical point during the spreading of the disease, after which the online interest in the virus starts declining. This is apparent especially for the cumulative cases and deaths, where one function is monotonous (increasing), while the other starts exhibiting a decrease after reaching a peak. Thus, said critical point should be identified in countries and regions with fewer cases to examine the possibility of using Google Trends data to nowcast the spread of COVID-19.

**Figure 4:**
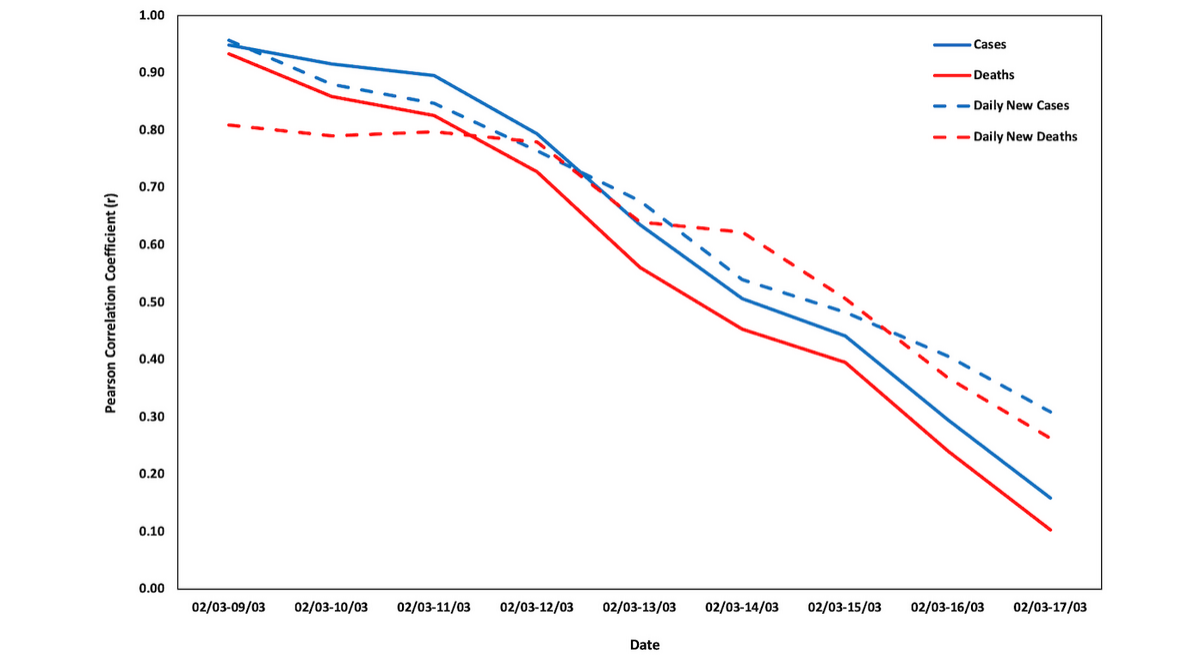
Changes in the Pearson correlation coefficients (*r*) for Italy.

**Figure 5:**
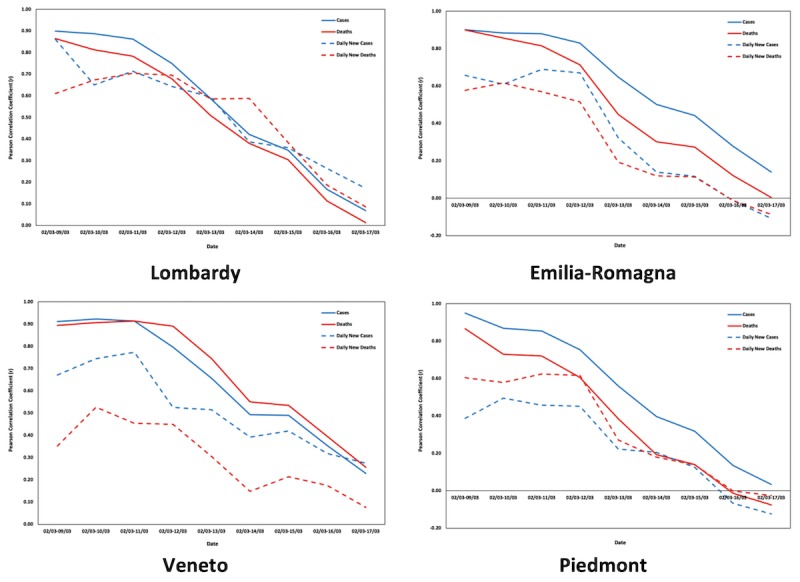
Changes in the Pearson correlation coefficients (*r*) for Lombardy.

Figures 4 and 5 depict the changes in the Pearson correlation coefficients (*r*) between Google Trends data and COVID-19 cases and deaths for the aforementioned time periods in Italy and Lombardy, respectively. Graphs for the respective changes in the Pearson correlation coefficients for the 20 Italian regions can be found in Multimedia Appendix 1.

Based on these results, it is suggested that regional nowcasting of COVID-19 is possible by simply monitoring Google Trends data until that critical point. This is of high significance if it is applied locally, as it could indicate the regions that will exhibit an increase in COVID-19 cases, thus increasing the preparedness of the health care systems, while, most importantly, taking the needed measures to minimize disease spreading.

**Figure 6:**
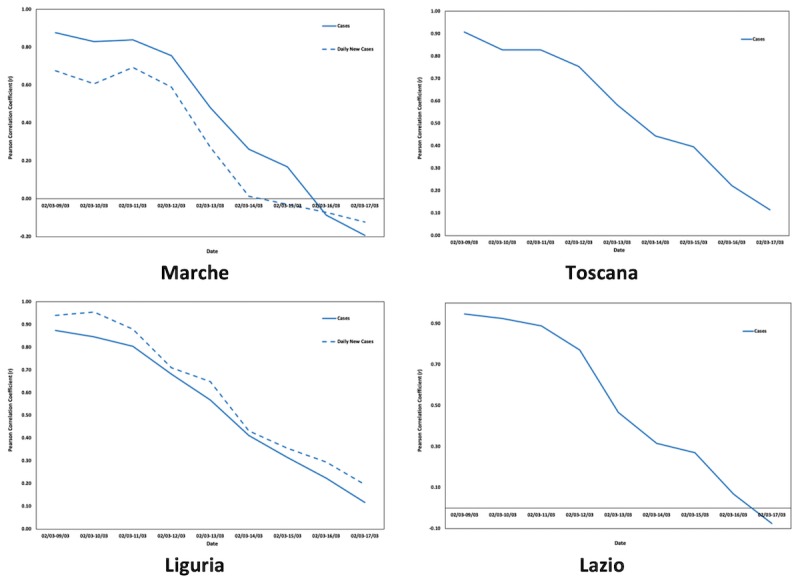
Changes in the Pearson correlation coefficients (*r*) for Spain.

**Figure 7:**
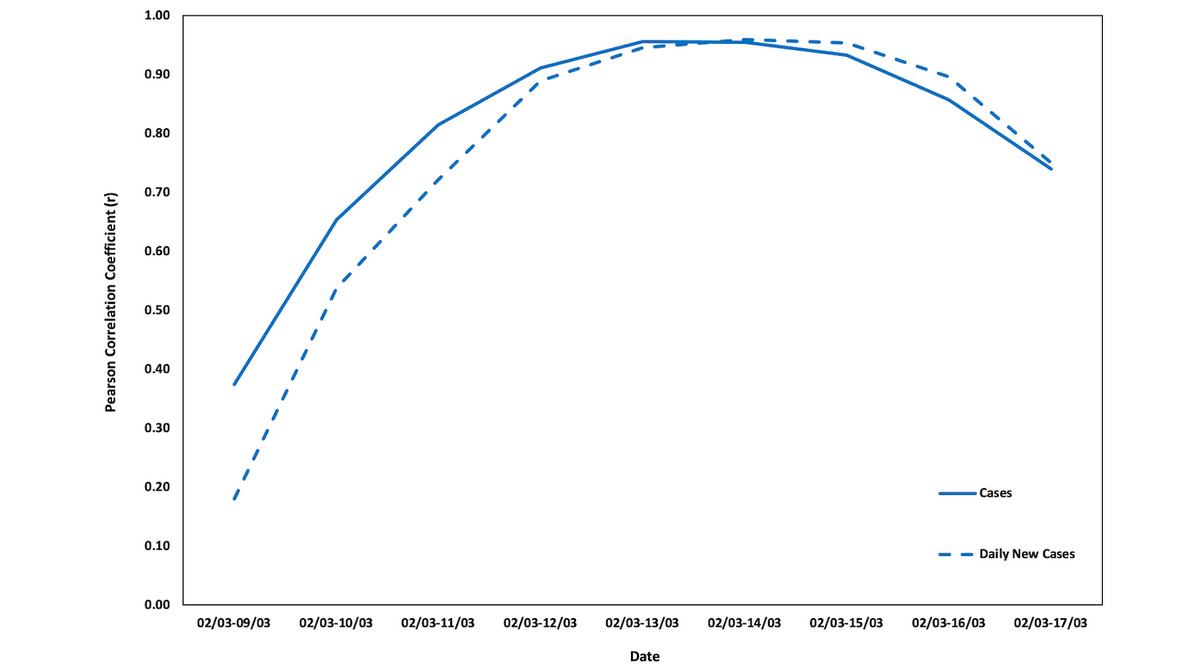
Changes in the Pearson correlation coefficients (*r*) for Germany.

**Figure 8:**
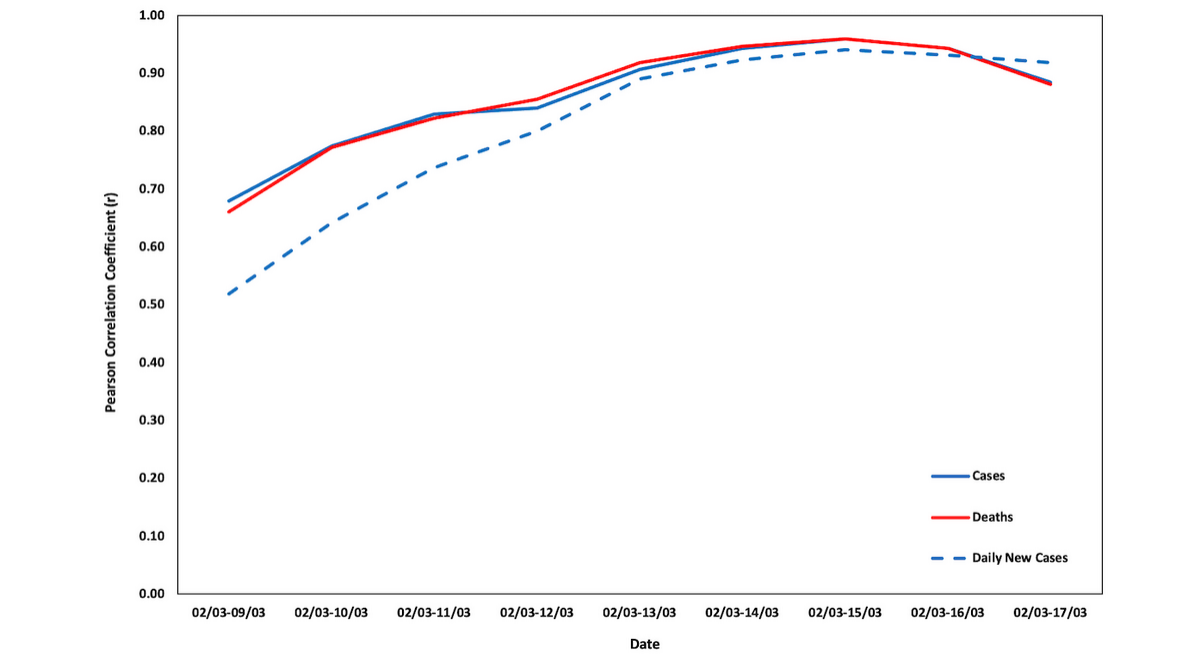
Changes in the Pearson correlation coefficients (*r*) for France.

In Europe, the countries experiencing the highest case and death counts (after Italy) are Spain, France, Germany, and the United Kingdom, with Spain being in an extremely difficult position with plane traffic being restricted and the army regulating local and regional movement. Thus, for the same time frames as for the Italian regions, the correlations between COVID-19 cases and deaths (where applicable) and the online interest in COVID-19 were calculated. Figures 6-8 depict the changes in the Pearson correlation coefficients for the selected time frames for Spain, Germany, and France.

For Spain, which is closely following Italy in COVID-19 cases and deaths, the Pearson correlation coefficient starts declining after March 13, 2020, which is when Spain’s death toll reached 100. In France, the curve still has an increasing trend (150 total deaths as of March 16, 2020), while Germany’s curve has started declining since March 15, which is when the country’s casualties from COVID-19 passed 10.

**Figure 9:**
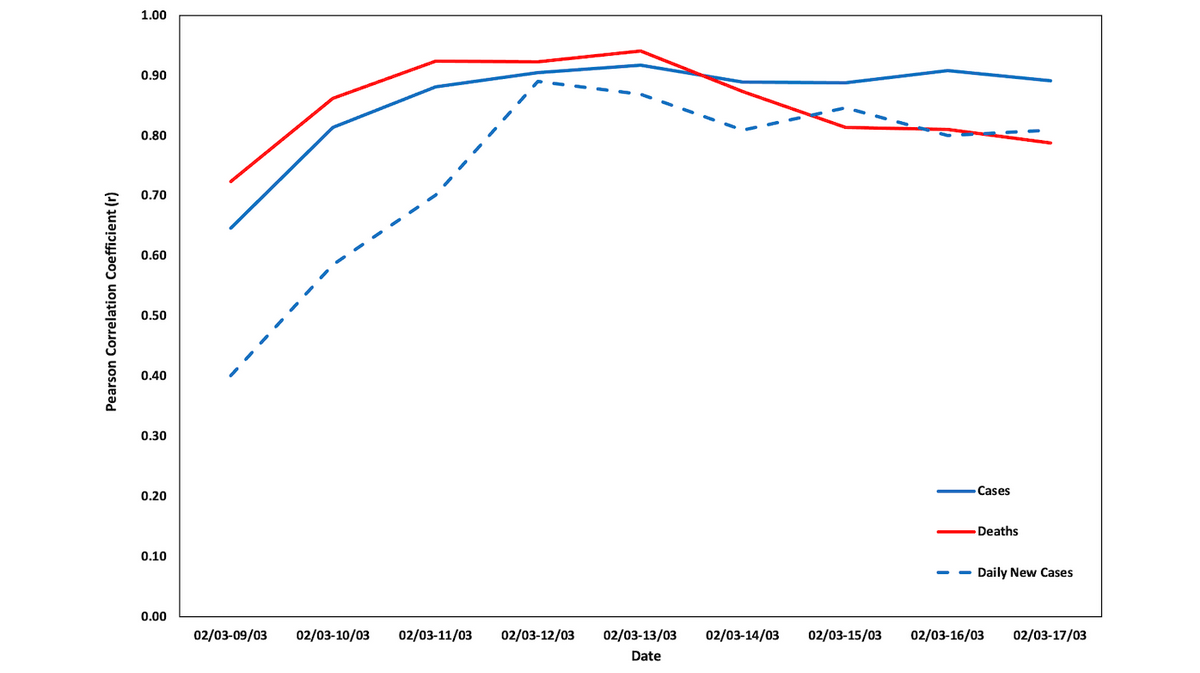
Changes in the Pearson correlation coefficients (*r*) for the United Kingdom.

Next, the most affected European country (ie, the United Kingdom with more than 10,000 cases) was selected to elaborate on the relationship between COVID-19 cases and deaths and the online interest in the topic. The United Kingdom followed a different approach than most European countries, by not taking preventive measures at an early stage. Figure 9 depicts the changes in the Pearson correlation coefficients for the same time frames selected previously. As is evident, the United Kingdom is still exhibiting high and statistically significant correlations (Table 5).

**Table 5:** Pearson correlation coefficients (*r*) between COVID-19 cases and deaths and Google Trends data for the United Kingdom.

Time Frames	Cases		Deaths		Daily Cases	
	*r*	*P* value	*r*	*P* value	*r*	*P* value
March 2-9	0.6470	.08	0.7241	.04	0.4008	.33
March 2-10	0.8144	.008	0.8629	.003	0.5863	.097
March 2-11	0.8811	<.001	0.9244	<.001	0.7021	.02
March 2-12	0.9053	<.001	0.9229	<.001	0.8907	<.001
March 2-13	0.9177	<.001	0.9408	<.001	0.8689	<.001
March 2-14	0.8896	<.001	0.8742	<.001	0.8091	<.001
March 2-15	0.8878	<.001	0.8145	<.001	0.8470	<.001
March 2-16	0.9083	<.001	0.8110	<.001	0.8010	<.001
March 2-17	0.8920	<.001	0.7878	<.001	0.8100	<.001

The relationship between COVID-19 cases and deaths shows an increasing trend over the examined period and stays high afterwards. Note that the United Kingdom had zero deaths March 2-4, 2020. The decrease is also evident in Table 5, which consists of the Pearson correlation coefficients and their significance, the latter also exhibiting increased rates as time moves forward, contrary to Italy, Spain, and all Italian regions.

Therefore, it is evident that a correlation between COVID-19 and Google Trends data exists, but the critical point, after which the online interest starts declining, should be identified in each individual case to proceed with regional nowcasting. Toward this direction, the data period should be shortened and applied to regions that have not yet been as severely affected. Google Trends provides a detailed regional break down for most countries, as well as real time and 1-hour interval data over the past week; this gives the opportunity of nowcasting users’ search patterns and online behavior toward the disease.

## Discussion

### Principal Findings

Infodemiology metrics and approaches are an integral part of health informatics, with the most popular sources being Twitter and Google [19,20], which have been successfully employed in the past to track and forecast outbreaks and epidemics (eg, Middle East respiratory syndrome [21], measles [22,23], Ebola [24,25], the swine flu [26], and the Zika epidemic [27,28]).

However, the case of the new coronavirus is somewhat different both in terms of the qualitative and quantitative approach than the previously examined epidemics. COVID-19 has been the subject of several controversial discussions. Since China’s first death report on January 11, 2020 [2], there have been several controversies regarding how China has handled the epidemic. There are ongoing debates as to whether there had been an attempt to hide the beginning of the outbreak, which became public by whistleblower Dr Li Wenliang who was reported dead as of February 7 due to COVID-19 complications [29]. There has been information about reporters being expelled from China as brought forward by New York Times reporter Amy Qin [30]. Most importantly though, there have been doubts about the accuracy of the data and results that the Chinese authorities and scientists have provided, with a much discussed incident being the announcement that “*Preliminary investigations conducted by the Chinese authorities have found no clear evidence of human-to-human transmission of the novel #coronavirus (2019-nCoV) identified in #Wuhan, #China*” [31].

However, the case of Italy, which is the country with the highest death toll and should perhaps be treated as the first case of what to expect from the virus spread, shows that the epidemic is far more serious than what the officials originally suggested, with a record daily death toll of 919 reported on March 27 [32] and total deaths slightly less than 10,000. Based on Italy’s data, many European countries acted fast in imposing measures for slowing down the spread of the disease, and the next 2-3 weeks could exhibit nonexponential curves in terms of daily casualties.

Toward the direction of finding new methods for nowcasting COVID-19 to increase the preparedness of health care systems, this study suggests that Google Trends data strongly correlates with COVID-19 cases and deaths worldwide and in the examined countries. Most importantly though, there is a critical point, after which the relationship’s strength (in almost all cases) monotonously decreases, even if the correlation remains statistically significant, with Italy having the sharpest downward curve.

### Limitations

This study has limitations. First, since the pandemic not only is ongoing but has not reached its peak yet, the data are limited; thus, the correlations are based on fewer observations, and the results are only preliminary and subject to change as we move forward. Second, only a few countries provided, at the time of writing, sufficient data for analysis or a regional break down of the cases and deaths. Third, only the interest in the ”Coronavirus (Virus)” Topic was explored, but future reports should also elaborate on more complicated search patterns, especially using the official name of the disease (ie, COVID-19) once it is used by a significant part of the population. Fourth, there are significant changes in cases, deaths, and rates even between 2 consecutive days in many regions and countries; even at the time of writing, the data can significantly vary from those at the time of retrieval.

### Conclusions

In line with previous studies that have indicated that Google Trends data can assist with the tracking and nowcasting of epidemics and outbreaks, the results of this paper show that online search traffic data are highly correlated with COVID-19 cases and deaths in the examined countries and regions. Furthermore, a critical point, up to which regions not severely affected exhibit the strongest relationship between Google and COVID-19 data, was identified. This suggests that focus should shift towards these regions to make full use of what real time data assessment can offer. The latter is essential for increasing the preparedness and responsiveness of local health institutions, which is the most important aspect in handling the current pandemic.

As of March 27, the center of the COVID-19 pandemic is the United States, with New York being the most affected, and it is imperative to perform similar analyses regionally, at state, metro, and city levels. Data from the disease spread and casualties in Europe will provide a better picture as to the characteristics of the virus as well as detailed data—both traditional and infodemiological—to estimate nowcasting models.

Despite the limited data availability at this stage of the pandemic, it is essential that all results are shared and rapid publications on the topic of infodemiology are accessible. Infodemiology results from various sources such as Google, Twitter, Facebook, or other social media are valuable variables in epidemiology. It is crucial to use such preliminary findings to build novel approaches that make use of real time data for the tracking and nowcasting of COVID-19.

## Appendix

Multimedia Appendix 1 Changes in the Pearson correlation coefficients (r) for the 20 Italian regions.

## Abbreviations

COVID-19: coronavirus disease
SARS: severe acute respiratory syndrome
