# Supplementary material for: Tracking COVID-19 in Europe: Infodemiology Approach
Source: JMIR Public Health Surveill. 2020 Apr 20;6(2):e18941. doi: 10.2196/18941 (PMC7173241; doi:10.2196/18941)
Supplement: Multimedia Appendix 1 [file publichealth_v6i2e18941_app1.pdf]

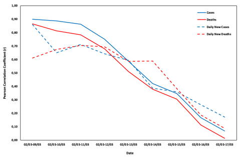

Lombardy

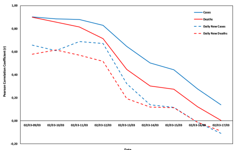

Emilia-Romagna

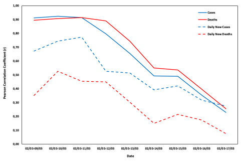

Veneto

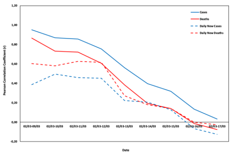

Piedmont

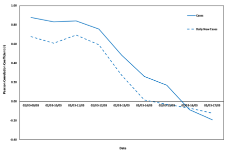

Marche

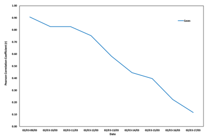

Toscana

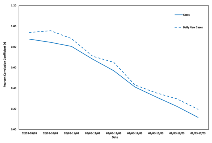

Liguria

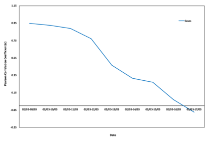

Lazio

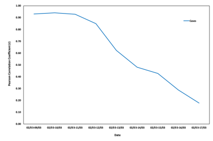

Friuli-Venezia Giulia

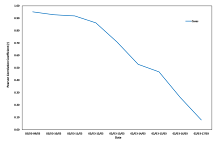

Campania

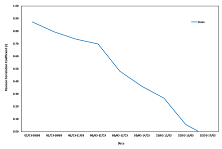

Trento

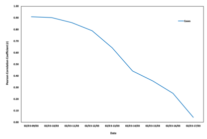

Apulia

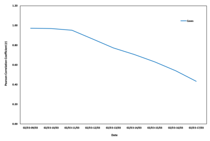

Sicily

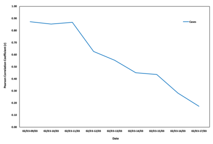

Abruzzo

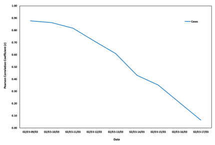

Umbria

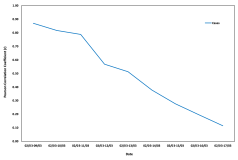

Aosta Valley

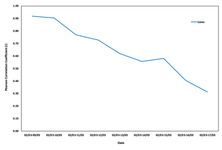

Sardinia

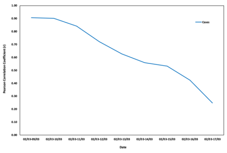

Calabria

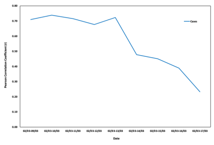

Molise

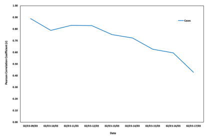

Basilicata
